# The prevalence of substance abuse and associated factors among male prisoners in Karachi jails, Pakistan

**DOI:** 10.1016/j.jtumed.2022.05.001

**Published:** 2022-05-28

**Authors:** Madiha Jamal, Shabana Waheed, Asma Shakoor

**Affiliations:** a General Dental Practitioner, Royal College of Surgeons of England, Cornwall, United Kingdom; b High Court of Sindh, Karachi, Pakistan; c Community & Preventive Dentistry Department, Institute of Dentistry, Combined Military Hospital- Lahore Medical College, National University of Medical Sciences, Lahore, Pakistan

**Keywords:** القنب, عقاقير غير مشروعة, الاضطرابات المتعلقة بالمخدرات, سجناء, انتشار, Cannabis, Illicit drugs, Prevalence, Prisoners, Substance-related disorders

## Abstract

**Objective:**

Substance abuse and cigarette smoking are now regarded as major public health problems. This study aims to determine the prevalence, risk profile, and associated sociodemographic factors of substance abuse among male prisoners in Karachi jails.

**Methods:**

This descriptive cross-sectional study was carried out among 600 male prisoners in Malir and Central Jails in Karachi. The principal investigator collected the data via one-on-one basis interviews, using a survey questionnaire consisting of the WHO ASSIST version 3, and structured demographic proforma. The data analysis regarding ever and current use of ten substances was done according to the standard instruction manual. A Chi-square test was applied to determine the association between categorical sociodemographic variables and current/ever substance use among prisoners. A p-value of <0.05 was considered statistically significant.

**Results:**

In this study, 97.1% of prisoners had a history of substance abuse at least once in their lifetime. For the current use of a substance, the majority of the prisoners fell into the low-risk category, except for tobacco for which 80.5% of prisoners were at moderate risk of use. 13%, 12.7%, and 9.5% of prisoners were at high risk for using opioids, cannabis, and tobacco, respectively. Several associations were found between the socioeconomic factors of the study subjects and their substance use.

**Conclusion:**

The study demonstrates a high prevalence and alarming current risk profile of substance abuse among prisoners of Karachi Jails. Considering the associated disease burden, substance abuse among prisoners should be considered a public health priority. Further exploration of associated and causative factors can help policymakers devise adequate measures for prevention and rehabilitation.

## الملخص

**أهداف البحث:**

يعتبر تعاطي المخدرات وتدخين السجائر من مشاكل الصحة العامة الرئيسية الآن. تهدف هذه الدراسة إلى تحديد مدى انتشار، وبيان المخاطر، والعوامل الاجتماعية والديموغرافية المرتبطة بتعاطي المخدرات بين السجناء الذكور في سجون كراتشي.

**طرق البحث:**

أجريت هذه الدراسة المقطعية الوصفية على 600 سجين ذكر في سجن مالير والسجن المركزي في كراتشي. قام الباحث الرئيس بجمع البيانات من خلال المقابلات الفردية، باستخدام استبانة من الإصدار الثالث لمسح "أسيست" من منظمة الصحة العالمية، بالإضافة إلى الخصائص الديموغرافية للسجناء. تم إجراء تحليل البيانات المتعلقة بتعاطي المخدرات سابقا أو حاليا لعشر مواد وفقا لدليل التعليمات القياسي. تم تطبيق اختبار مربع كاي لتحديد الارتباط بين المتغيرات الاجتماعية الديموغرافية المصنفة وتعاطي المخدرات سابقا أو حاليا بين السجناء. اعتبرت قيمة الاحتمال الأقل من 0.05 ذات دلالة إحصائية.

**النتائج:**

في هذه الدراسة، 97.1٪ من السجناء لديهم تاريخ من تعاطي المخدرات مرة واحدة على الأقل في حياتهم. بالنسبة للاستخدام الحالي لمادة ما، فإن غالبية السجناء يندرجون في فئة منخفضة المخاطر، باستثناء التبغ الذي كان 80.5٪ من السجناء معرضين لخطر متوسط من استخدامه. كان 13٪ و 12.7٪ و 9.5٪ من السجناء معرضين لخطر كبير لاستخدام المواد الأفيونية والقنب والتبغ، على التوالي. تم العثور على العديد من الارتباطات بين العوامل الاجتماعية والاقتصادية للسجناء وتعاطيهم المخدرات.

**الاستنتاجات:**

توضح الدراسة الانتشار الواسع والمخاطر الحالية المقلقة لتعاطي المخدرات بين سجناء سجون كراتشي. بالنظر إلى عبء الأمراض المرتبطة بتعاطي المخدرات، يجب اعتبار تعاطي المخدرات بين السجناء من أولويات الصحة العامة. يمكن لمزيد من الاستكشاف للعوامل المرتبطة والمسببة أن تساعد صانعي القرار على استنباط تدابير مناسبة للوقاية وإعادة التأهيل.

## Introduction

Drug addiction is a chronically relapsing disorder, characterized by the compulsive use of addictive substances despite adverse consequences to the individual and society.^1^ Frequent substances of abuse include licit and illicit drugs, such as alcohol, cannabis, cocaine, amphetamine-type stimulants, hallucinogens, opioids, and other designer drugs.^2^ The production, sale and use of illicit drugs are prohibited in most countries. While licit drugs are legally available by medical prescription in the jurisdiction in question, over-the-counter such as sedative and sleeping pills, or commercially available, e.g., all forms of tobacco.^3^

Substance abuse and cigarette smoking are now regarded as major public health problems.^4^ According to the 2017 status report, the globally estimated prevalence of episodic alcohol use and daily tobacco smoking among the adult population was 18.4% and 15.2%, respectively.^2^ Communicable diseases such as hepatitis B, hepatitis C, and HIV are continuously adding up to the global burden of diseases, because of the intravenous illicit drug abuse.^5^ Furthermore, opium, tobacco, and alcohol users have a higher risk for esophageal squamous cell carcinoma.^6^ The use of Tobacco and amphetamine-type stimulants is a known risk factor for myocardial infarction and other cardiovascular diseases like cardiomyopathy.^7^^,^^8^ Mental health problems are also a concern, as the use of cannabis is a risk factor for psychosis.^9^ Substance abuse in non-fatal doses can result in morbidity and serious health consequences, thus affecting the quality of life.^10^ In 2016, approximately 18.45 million and 21.89 million healthy years of life were estimated to be lost due to alcohol abuse and drugs abuse disorders, respectively.^10^ In addition, drug abuse is associated with a multitude of social, psychological, and economical sequelae. The most serious outcome of alcohol and drug abuse is death, which respectively accounted for 0.26% and 0.28% of all-cause mortality on the global scale in 2016.^10^

Studies have shown that drug abuse within prisons is common.^11^ In low- and middle-income countries, approximately 30–56% of the imprisoned people, predominantly males, use illicit drugs.^11^ Whereas the prevalence is higher in high-income countries, particularly among female prisoners.^11^ According to a meta-analysis of 14,527 prisoners of low socio-economic countries like Pakistan, alcohol and drug abuse disorders were respectively twice and six times more prevalent among prisoners as compared to the general population.^12^ These disorders increase the risk of a range of adverse outcomes among prisoners, including mental health problems,^13^ infectious diseases,^14^ recidivism,^15^ and death.^16^ Substance abuse disorders also significantly increase the rate of all-cause mortality among prisoners, and the prediction of accidents, suicide, and homicide increases after their release from prison.^17^ Several associations were found between the socioeconomic factors of the study subjects and their substance use.^17^

Little is known about the different aspects of substance abuse among prisoners in Pakistan, as only a handful of studies are available.^18^, ^19^, ^20^ This study aims to determine the prevalence and risk profile of substance abuse among male prisoners in Karachi jails. This study also intends to assess the association of substance abuse with sociodemographic variables of prisoners.

## Materials and Methods

### Study design and participants

This descriptive cross-sectional study was carried out among prisoners in Malir and Central Jails, Karachi, from April to September 2018. Male prisoners who were willing to participate and between 18 and 60 years old were included in the study. Female prisoners, and those prisoners who had severe communication difficulties or mental/behavioral disturbances were excluded. The sample size of 343 was calculated by the Open Epi calculator, using a prevalence, of drug users in prisons, at 64.7%,^21^ at a 5% margin of error, and 95% confidence level.

### Data collection

To reduce missing observation bias, the sample size was upsized, and data was collected from 600 prisoners. Convenience sampling technique was used, and the data was collected using one-on-one basis interviews. These interviews were conducted after briefly explaining the relevant details, ensuring confidentiality, and obtaining written consent.

The questionnaire used by the principal investigator consisted of WHO Alcohol, Smoking and, Substance Involvement Screening Test (ASSIST) version 3.0, and a structured proforma for recording the demographic variables of the subject. Scoring of the risk profile assessment was done according to the instruction manual of the ASSIST tool.^22^ Drug abuse data was collected into two broadly classified terms; Ever used i.e., substances the individual used even if only once, and current use i.e., a substance that was used in the past three months. Data regarding Ever-use of substance was recorded as a binary variable (never/yes). Data for the current use of ten different substances was collected using items 2–7 of the ASSIST questionnaire. Each question had a set of responses to choose from, and each response from questions 2 to 7 has a numerical score. At the end of the interview, according to the responses of the subject, the scores of each substance were added together from items 2 through 7 i.e., tobacco, alcohol, cannabis, cocaine, amphetamine-type stimulants, inhalants, sedatives, hallucinogens, opioids, and ‘other’ drugs.

As laid out in the ASSIST questionnaire, each subject had 10 risk scores for each of the above-mentioned substances. The risk score for the current use of Tobacco ranged between 0 and 31. The risk score for alcohol, cannabis, cocaine, amphetamine-type stimulants, inhalants, sedatives or sleeping pills, hallucinogens, opioids, and other drugs ranged between 0 and 39.^22^ Based on the score achieved, each subject was classified into low, moderate, or high-risk individual as demonstrated in Table 1.^22^

**Table 1:** Description about Scoring criteria.

Risk level	Alcoholic beverages	Tobacco and other substances
Low	0–10	0–3
Moderate	11–26	4–26
High	27+	27+

Sociodemographic profiles collected via questionnaire included age (noted as a continuous variable), gender (male or female), marital status (currently married or unmarried), family system (nuclear or joint), residential information (Rural or Urban), and employment status before imprisonment (employed, unemployed, or self-employed). Education level was collected as a total number of years of a full education, categorized according to Kuppuswamy's scale^23^ into five categories as; No education (0 years or those who never attended school), Primary school (1–5 years), Middle/full abbreviation (HSC) (6–10 years), Intermediate (11–12 years), Graduate (13–16 years), and Postgraduate (16+ years).

The monthly family income was measured as a continuous variable in multiples of thousands and then categorized using Kuppuswamy's scale. The scale is based on Indian income, so it was converted in this study to a Pakistani income and was categorized into eight cut off values^23^ as; <3, 3–9, 9–15, 15–23, 23–30, 30–60, or above 60. Occupation of the prisoners was classified into seven categories according to Kuppuswamy's scale^23^ as; Unemployed (able or unable to work), Unskilled worker (vendors, bus conductor, dishwasher), Semi-skilled (driver, cable operator, key maker), skilled worker (electrician, baker, business, computer hardware technician), clerical worker (general store owner, farmer), semi-professional (research associate, nurse), and professional (research supervisor, teacher, human resource officer).

### Data analysis

Statistical package for social sciences (SPSS) version 26 was used for data entry and statistical analysis. Using descriptive analysis techniques, all continuous variables were computed as means and standard deviations, and categorical variables were described as frequency and percentage. In accordance to meeting the assumptions, Chi-square or Fischer Exact Tests were applied to determine the association between categorical sociodemographic variables and current/ever substance use among prisoners. To enhance the understandability of results, categories with too little data were combined while applying Chi-square analysis. Independent sample t-test was applied to compare the mean age of ‘never users’ with ‘ever users’ of drugs. A p-value of ≤0.05 was considered statistically significant, and only significant results were reported in the results section.

## Results

This study included data from 600 male prisoners from Karachi Jails through one-to-one interviews. The response rate of completed surveys was 100%. The mean age (± standard deviation) of the study subjects was 27.2 (±7.84) years old. About 54.8% of the subjects were currently married and 58.2% of them were living in a joint family system. About half of the study participants (50.2%) had never received a formal education, and about three quarters of them (75.8%) were unemployed before imprisonment. The majority of them prisoners (42.5%) were semi-skilled workers by occupation. About 35.7% reported that they had an approximate monthly family income of 9000–15,000 Pakistani Rupees before imprisonment. The details of various socio-demographic factors of the study subjects included in this study are given in Table 2.

**Table 2:** Sociodemographic characteristics of male prisoners (n = 600).

Characteristic		Mean	Standard deviation
Age in years		27.2	7.84
	Sub-category	Frequency (N)	Percentage (%)
Marital status	Currently married	329	54.8
	Currently unmarried	271	45.2
Family system	Nuclear	251	41.8
	Joint	349	58.2
Residence	Rural	542	90.3
	Urban	58	9.7
Educational status	No Education	303	50.5
	Primary	85	14.2
	Middle	182	30.3
	Intermediate	18	03
	Graduate	08	1.3
	Postgraduate	04	0.7
Employment before imprisonment	Unemployed	455	75.8
	Employed	11	1.8
	Self-employed	134	22.3
Occupation	No employment	117	19.5
	Unskilled	162	27
	Semi-skilled	255	42.5
	Skilled	49	8.2
	Clerk/Farmer	10	1.7
	Semi-professional	03	0.5
	professional	04	0.7
Monthly family income	<3	15	2.5
	3–9	141	23.5
	9–15	214	35.7
	15–23	105	17.5
	23–30	61	10.2
	30–60	45	7.5
	>60	19	3.1

A large proportion of prisoners (n = 550, 91.7%) reported that they had used one of the given drugs at least once in their life. The risk assessment profile of current substance abuse among prisoners showed that Tobacco had the highest risk score of abuse (15.99 ± 8.14), followed by Cannabis (8.1 ± 11.46) and Opioids (5.67 ± 12.19). Risk classification showed that Opioid abuse had the largest percentage in the high-risk category i.e., among 13% of the prisoners, followed by Cannabis and Tobacco among 12.7% and 9.5% of prisoners, respectively. However, among the medium-risk of substance abuse, Tobacco use was the highest (80.5%) followed by Cannabis (35.2%). Details of risk categorization of current substance abuse among prisoners for all the ten substances are shown in Table 3.

**Table 3:** Risk assessment profile of current substance use among prisoners (n = 600).

Substance of abuse	Risk score		Risk category		
	Mean	Standard deviation	Low	Medium	High
			N (%)	N (%)	N (%)
Tobacco	15.99	8.14	60 (10)	483 (80.5)	57 (9.5)
Alcohol	1.58	3.66	588 (98)	5 (0.8)	7 (1.2)
Cannabis	8.10	11.46	313 (52.2)	211 (35.2)	76 (12.7)
Cocaine	0.46	2.87	576 (96)	21 (3.5)	3 (0.5)
Amphetamine type stimulants	1.16	5.63	564 (99.6)	23 (0.3)	13 (0.1)
Inhalants	0.08	1.49	598 (99.7)	01 (0.2)	01 (0.2)
Sedatives	1.11	4.59	549 (91.5)	43 (7.2)	8 (1.3)
Hallucinogens	0.02	0.27	591 (99.8)	01 (0.2)	00 (0.0)
Opioids	5.67	12.19	452 (75.3)	70 (11.7)	78 (13)
Others	0.01	0.25	598 (99.7)	1 (0.2)	1 (0.2)

**Figure 1:**
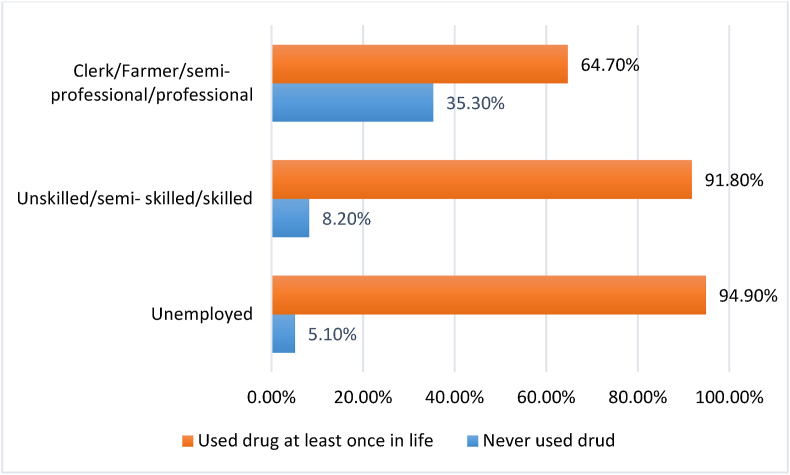
Ever drug use among prisoners classified according to their occupation before imprisonment.

Upon Determining the association of sociodemographic factors of prisoners with their history of ever drug use indicated that occupation was the only significant sociodemographic determinant of ever drug use among prisoners (p < 0.01). About 94.9% of the unemployed and 91.8% of the unskilled and skilled prisoners had a history of drug use at least once in their lifetime. Whereas the history of ever drug use was positive among 64.7% of the prisoners who were farmers/semi-professionals and professionals by occupation (Figure 1). No other sociodemographic factors demonstrated significant association with the history of ever drug use among prisoners (Table 4).

**Table 4:** Association of sociodemographic factors of prisoners with history of ever drug use.

Characteristic		Never mean (SD)	Yes mean (SD)	t-statistic	p-value
Age	Age in years	27 ± 8.94	27.22 ± 7.74	0.19	0.85
	Sub-category	Never N (%)	Yes N (%)	X^2^-statistic	p-value
Marital status	Currently married	26 (9.6%)	245 (90.4%)	1.03	0.37
	Currently unmarried	24 (7.3%)	305 (92.7%)		
Family system	Nuclear	20 (8.0%)	231 (92.0%)	0.08	0.88
	Joint	30 (8.6%)	319 (91.4%)		
Residence	Rural	7 (12.1%)	51 (87.9%)	1.06	0.31
	Urban	43 (7.9%)	499 (92.1%)		
Educational status	No Education	26 (8.6%)	277 (91.4%)	3.27	0.2
	Primary/Middle	19 (7.1%)	248 (92.9%)		
	Intermediate/Graduate/Postgraduate	5 (16.7%)	25 (83.3%)		
Employment before imprisonment	Unemployed	34 (7.5%)	421 (92.5%)	2.19	0.3
	Employed	01 (9.1%)	10 (90.9%)		
	Self-employed	15 (11.2%)	119 (88.8%)		
Occupation	Unemployed	6 (5.1%)	111 (94.9%)	17.77	<0.01∗
	Unskilled/semi-skilled/skilled	38 (8.2%)	428 (91.8%)		
	Clerk/Farmer/semi-professional/professional	6 (35.3%)	11 (64.7%)		
Monthly Family Income	<9	11 (7.1%)	145 (92.9%)	5.28	0.15
	9–15	15 (7%)	199 (93.0%)		
	15–30	14 (8.4%)	152 (91.6%)		
	>30	10 (15.6%)	54 (84.4%)		

∗p-value computed by Chi-square/Fischer Exact test analysis, significant at ≤0.05.

The association between sociodemographic factors and the risk profile of current abuse of substance among prisoners showed that Tobacco current use risk profile among prisoners was significantly associated with their occupation (p = 0.002), and their place of residence (p = 0.04). The type of subjects' occupation was also a significant associative factor of current use of alcohol (p = 0.48). Details of significant sociodemographic factors associated with their current risk profile of substance abuse among prisoners are demonstrated in Table 5.

**Table 5:** Association of sociodemographic factors with current substance use risk among prisoners.

Character	Risk category N (%)			
	Low	Medium	High	p-value
**Occupation**	**Tobacco**			
Unemployed	6 (5.1%)	101 (86.3%)	10 (8.5%)	0.002∗
Unskilled/Semi/Skilled	47 (10.1%)	372 (79.8%)	47 (10.1%)	
Semi-professional/Professional	7 (41.2%)	10 (58.8%)	0 (0.0%)	
**Occupation**	**Alcohol**			
Unemployed	112 (95.7%)	3 (2.6%)	2 (1.7%)	0.048∗
Unskilled/Semi/Skilled	460 (98.7%)	2 (0.4%)	4 (0.9%)	
Semi-professional/Professional	16 (94.1%)	0 (0.0%)	1 (5.9%)	
**Family system**	**Cocaine**			
Nuclear	176 (70.1%)	39 (15.5%)	36 (14.3%)	0.02∗
Joint	276 (79.1%)	31 (8.9%)	42 (12.0%)	
**Residence**	**Tobacco**			
Rural	51 (9.4%)	435 (80.3%)	56 (10.3%)	0.04∗
Urban	9 (15.5%)	48 (82.8%)	01 (1.7%)	

∗p-value computed by Chi-square/Fischer Exact test analysis, significant at ≤0.05.

## Discussion

This study showed that the majority of the prisoners in Karachi jails fell into the low-risk category of current substance abuse, except for tobacco which 80.5% of prisoners were at moderate risk of abuse. 13%, 12.7%, and 9.5% of prisoners were at high risk for using opioids, cannabis, and tobacco, respectively. Also, the majority of the study subjects (91.7%) had a history of substance abuse at least once in their lifetime.

A previous study of 700 inmates in central jail, Faisalabad in Pakistan reported that 96.43% of jail inmates had a risk of illicit drug abuse inside jail.^18^ The Overall prevalence of substance abuse among 2400 prisoners of the Central prison in Peshawar, Pakistan was estimated to be 72.8%, in 2009.^19^ A resembling trend of high prevalence reaching 86.6% of lifetime substance abuse was reported among Nigerian prisoners in 2020.^24^

In a survey of 336 prisoners from a jail in Ethiopia in 2018, the overall prevalence of self-reported substance abuse disorder was 55.9%.^25^ A similar study among Swedish prisoners in 2018, estimated the prevalence of lifetime abuse of illicit substances to be 37.1%.^26^ A meta-analysis of the prevalence of substance use among prisoners of low socioeconomic countries estimated that approximately one-fourth of the imprisoned population use illicit drugs.^27^ Another meta-analysis of 23 studies of 14,527 prisoners belonging to 13 low socioeconomic countries estimated the one-year pooled prevalence of alcohol and drug use disorder to be 3.8% and 5.1%, respectively.^12^

The variations between previous studies and the current study can be attributed to the geographical variations and methodological approaches used. A meta-analysis in 2019 reported geographical variation as a cause of heterogeneity in substance abuse prevalence.^12^ This meta-analysis reported that alcohol use disorders were highly prevalent among southeast Asian countries as compared to eastern Mediterranean areas of Europe.^12^ The results highlight the importance of exploring the data on substance abuse among various geographical regions to enhance preventive measures effectively.

In the current study, the occupation of prisoners was the only demographic variable significantly associated with ever substance use. Additionally, the current substance abuse was associated with lack of education, unemployment, unskilled and skilled occupations, living in a joint family system, and living in urban areas. Similarly, A previous study among 2400 jail inmates of central prison Peshawar reported that the majority (71.9%) of the substance abusers were illiterate.^19^ A survey of Ethiopian prisoners reported the absence of social support and urban residence to be positive associative factors of substance use among inmates.^25^ Similarly, unemployment had a significant impact on the high prevalence of the lifetime and current psychoactive substance abuse among prisnors in Nigeria.^24^

This study demonstrated that male gender, broken family system, and parental history of drug use as significant factors of current and lifetime substance abuse among prisoners.^24^ However, a self-reporting survey among female Spanish prisoners showed a prevalence of lifetime substance of 52.0%, with no significant association to marital status, level of education, and pre-imprisonment employment.^28^ Also, no significant associations were found between the place of residence, employment status, age, education level, and marital status with either current or ever use of a substance among Nigerian prisoners.^24^ The variation of reported results can be attributed to the heterogeneity of geographical and sociocultural factors among populations of referred studies. Identifying such factors can help the related administration in implementing adequate policies, especially for those at high risk of substance abuse, in resource deficient areas.

Age and marital status of the prisoners had no association with ever or current use of substances among prisoners in the current study. However, a previous study in Pakistan had reported a major occurrence of substance use among prisoners 40–50 years of age.^19^ Similarly, a review article in 2019, which analyzed 17 studies on inmates, concluded that older prisoners were more likely to abuse alcohol than the younger ones.^29^ Additionally, a high prevalence of illicit substance abuse was observed among young female Spanish inmates.^28^ An analysis of medico-legal files of 380 male prisoners of 13 jails in Switzerland estimated that 50% of younger prisoners, compared to 24.2% of older prisoners, had abused illicit drugs at least once in a lifetime.^26^ Interestingly, the prevalence of substance abuse among younger and older prisoners varied according to the substance of abuse. For example, the current cannabis abuse was higher (10%) among younger prisoners as compared to the older offenders (3.2%).^26^ Such differences, in the relation of age of substance abuse, could be attributed to the age classification used. In previous studies, participants were categorized into young and old, whereas in this study we used age as a continuous variable, without categorization.

The implication of this study is limited due to its cross-sectional design. Due to limited resources, this study is carrying another limitation of using interview-based screening of substance abuse rather than adopting biochemical diagnostic methods. This study includes a sampled population from the jails of only one city in Pakistan, rendering the generalization of results difficult owing to the sociocultural, ethnic, and religious biases. Compared to currently published literature, the current study has several important strengths and contributions to the existing knowledge owing to its large sample size from both prisons in Karachi. Another strength of this study is exploring risk factors of both lifetime and current consumption of drugs while using a standard WHO Questionnaire, which can help in comparison and generalization internationally.

## Conclusion

The study demonstrates a high prevalence and alarming current risk profile of substance abuse among prisoners of Karachi Jails. Considering the associated disease burden, substance abuse among prisoners should be considered a public health priority. Further exploration of associated and causative factors can help policymakers devise adequate measures for prevention and rehabilitation.

## Recommendations

The findings of this study are reflecting an unmet need of prisoners, which should be intervened at priority as a public health issue instead of a criminal justice approach. This study implies a dire need for a comprehensive review of our criminal justice system, to develop a risk assessment system, and to build effective coordination with rehabilitation centers.

## Source of funding

This research did not receive any specific grant from funding agencies in the public, commercial, or not-for-profit sectors.

## Conflict of interest

The authors have no conflict of interest to declare.

## Ethical approval

This study was reviewed and approved by the ethical review committee of Dow University of Health Sciences, Karachi Reference Number IRB-801/DUHS/Approval/2016/338 (8th December 2016).

## Authors contributions

MJ conceived and designed the study, conducted research, collected and organised the data and wrote the initial draft of the manuscript. SW conceptualization, conducted the literature review, analysed and interpreted the data. Wrote final draft of the manuscript. AS, participated in project administration, designed methodology, reviewed the manuscript and approved its final revision. All the authors have critically reviewed and approved the final draft and are responsible for the accuracy and integrity of the content and similarity index of the manuscript.
